# Creating and Maintaining a “Climate-Smart” Emergency Department: A Scoping Review of Current Progress and Future Potential

**DOI:** 10.5811/westjem.50597

**Published:** 2026-05-03

**Authors:** Lea Moujaes, Kayla Iuliucci

**Affiliations:** Johns Hopkins University School of Medicine, Department of Emergency Medicine, Baltimore, Maryland

## Abstract

**Introduction:**

Climate change represents one of the most significant global health threats, with emergency departments (ED) serving as frontline responders to climate-related health emergencies. While EDs are major contributors to healthcare’s environmental footprint and critical responders for climate disasters, no comprehensive review has examined sustainability and climate-resilience initiatives specifically implemented in ED settings.

**Methods:**

We conducted a scoping review examining literature on sustainable and climate-resilient measures in EDs. Comprehensive searches of PubMed, Scopus, and Embase were performed from inception through November 2024, using terms related to EDs combined with sustainability and climate-resilience concepts. Two reviewers independently screened papers, with inclusion criteria requiring ED-specific focus and concrete sustainability or resilience interventions.

**Results:**

Seven studies met inclusion criteria, representing diverse geographic contexts. Three addressed sustainability interventions including waste reduction, sustainable procurement, device reprocessing, and renewable energy adoption. Case examples demonstrated co-benefits, such as 31% reduction in ambulance carbon dioxide emissions and $3 million savings from device reprocessing programs. All studies described resilience interventions encompassing disaster preparedness, surge capacity, infrastructure continuity, and clinical protocols. However, significant gaps were identified: Only 13–20% of hospitals in surveyed countries had disaster plans, and no studies documented fully operational climate-smart EDs. Global frameworks were referenced but not operationalized in ED settings.

**Conclusion:**

There is a limited body of peer-reviewed studies that describe measures to close the implementation gap between current climate science and operational practices in EDs. Despite extensive policy recommendations and demonstrated benefits, no studies have described any existing programs. Emergency medicine requires translation of conceptual frameworks into measurable interventions, standardized outcome measures, and systematic implementation of climate-smart healthcare practices.

## INTRODUCTION

Climate change is increasingly recognized as one of the most prominent global health threats of the 21st century, with mounting evidence that its effects are already disrupting healthcare systems worldwide.^1^ Emergency departments (ED) are on the frontlines of this crisis, witnessing firsthand the consequences of rising global temperatures, extreme weather events, and deteriorating air and water quality. These climate-related stressors are driving measurable increases in ED patient volumes, particularly during periods of extreme heat, wildfires, flooding, and other climate disasters. Such events lead to spikes in heat illnesses, exacerbations of chronic diseases, trauma, and mental health emergencies disproportionately affecting vulnerable populations including the elderly, children, individuals with pre-existing health conditions, and communities that are socioeconomically disadvantaged.^2^

At the same time, the healthcare sector itself contributes significantly to the climate crisis, accounting for an estimated 4–5% of global greenhouse gas (GHG) emissions.^3^ In the United States, the healthcare industry has been estimated to contribute about 8.5–10% of the nation’s GHG emissions.^4^,^5^ In response to this paradox, health systems around the world have begun to implement mitigation and adaptation strategies aimed at reducing their environmental impact while preparing for climate-related disasters. These efforts include integrating the World Health Organization (WHO) framework for climate-resilient health systems, adopting renewable energy sources, decarbonizing supply chains, reforming wasteful procurement practices, and establishing institutional alliances committed to environmental sustainability. Several national health systems have also launched climate action plans, introduced emissions tracking metrics, and invested in greener infrastructure.^6^ Further, remarkable developments too recent to have been described in peer-reviewed literature have been initiated. Chief among these is the opening of an “all-electric” hospital that is fully renewably powered, at the University of California Irvine, in Irvine, CA.^7^ And the British National Health Service (NHS) is systematically placing new, renewably powered ambulances into service, as current petroleum-powered ambulances are retired from service. This is but one component of Great Britain’s initiative to deliver a “net zero” NHS.^8^

Despite these promising developments, most sustainability initiatives remain focused at the hospital system level, with limited documentation of climate-conscious interventions within specific clinical departments. Emergency departments, which operate around the clock and are among the most resource-intensive units in healthcare, have largely been left out of this conversation. Yet EDs are uniquely positioned to model both mitigation and adaptation strategies. Not only do they absorb the immediate impacts of climate-related health emergencies, but they also produce considerable waste, consume large amounts of energy (especially if the GHG emissions from ambulances that deliver patients to the ED are considered), and function as key decision-making nodes within hospitals. Given this dual role, EDs represent critical leverage points for climate action within the healthcare sector. However, to date there has been no comprehensive review of the strategies EDs have implemented to reduce their environmental footprint or to enhance their climate resilience. A better understanding of these efforts is urgently needed to inform clinical operations, policy development, interdisciplinary collaboration, and emergency preparedness planning.

**Population Health Research Capsule**

What do we already know about this issue?

*Climate change is increasing ED demand, and healthcare delivery contributes significantly to greenhouse gas emissions*.

What was the research question?

*What sustainability and climate-resilience interventions have been implemented in EDs?*

What was the major finding of the study?

*Only seven studies met criteria; no EDs reported fully implemented climate-smart programs*.

How does this improve population health?

*This scoping review reveals a persistent implementation gap, informing efforts to reduce ED emissions and strengthen preparedness for climate-related health threats*.

We sought to address this gap by systematically identifying and analyzing the sustainability and climate-resilience initiatives that have been implemented in EDs and described in peer-reviewed literature. Specifically, we aimed to map the range of environmental interventions described in the literature, assess the use of global climate- and disaster-resilience frameworks in the ED setting, and highlight opportunities for future development, collaboration, and standardization in ED-based climate action.

## METHODS

### Study Design

In this scoping review we examined the literature on implementation of sustainable and climate-resilient measures in the ED. We conducted comprehensive searches of three electronic databases: PubMed; Scopus; and Embase from inception through November 2024. Only English-language studies were included. The search strategy combined terms related to EDs (eg, “emergency department,” “emergency room,” “ED,” “ER”) with sustainability and climate resilience terms (eg, “sustainability,” “climate resilience,” “climate adaptation,” “environmental sustainability,” “green healthcare,” “carbon footprint”). We used Boolean operators to combine search terms appropriately.

### Study Selection and Screening

We imported search results into Covidence software (Veritas Health Innovations, Ltd, Melbourne, Victoria, Australia) for screening and management, completed by December 2024. Two reviewers independently screened titles, abstracts, and full-text papers. We included studies that were specifically focused on EDs and addressed specific sustainability and resilience tactics or interventions. We excluded studies conducted outside ED settings or those that did not address concrete techniques for resilience and sustainability implementation. Initial screening yielded 217 papers for full-text review. Following application of inclusion and exclusion criteria, seven papers met criteria for final inclusion in the review.

### Data Extraction

Data extraction focusing on qualitative concepts was completed by February 2025 using dual review methodology. Given the exploratory nature of this scoping review, we did not employ strict systematic review protocols such as PRISMA guidelines. Data were extracted and compiled in Microsoft Excel (Microsoft Corporation, Redmond, WA) for analysis.

## RESULTS

### Study Selection and Characteristics

Seven studies met the inclusion criteria and were included in the review.^9^–^15^ They represented a mix of systematic and scoping reviews, narrative and conceptual analyses, a primer introducing a framework for climate-smart EDs, a clinical-tool development paper, and an international policy perspective. Collectively, the studies spanned diverse geographic contexts, including the U.S.,^11^,^13^,^15^ low- and middle-income countries in multiple regions,^10^ a continent-wide review focused on Africa,^14^ and papers addressing international or cross-regional perspectives.^9^ The EDs were described broadly as critical, high-volume hospital entry points and frontline disaster-response nodes.^10^,^11^,^13^,^14^ However, few studies reported specific ED size or volume data; most focused on system-level vulnerabilities, facility preparedness, or conceptual frameworks.^9^–^11^,^13^,^14^ Vulnerable populations that were consistently identified across studies included children, older adults, women and pregnant persons, people with chronic disease, and socially marginalized groups.^9^,^11^,^13^,^14^

### Sustainability Interventions

Three of the included studies addressed sustainability and mitigation strategies relevant to EDs.^9^,^12^,^13^ Reported operational domains encompassed waste segregation and reduction, sustainable procurement, device reprocessing, energy and water efficiency, green transportation, and renewable energy adoption.^12^,^13^ Case examples demonstrated potential for both environmental and financial co-benefits. For instance, a U.S. emergency medical services (EMS) fleet conversion achieved a 31% reduction in ambulance CO_2_ emissions over one year, while single-use device reprocessing programs at one hospital saved $3 million and diverted nearly 19,000 pounds of waste in the same period.^12^ Health-sector recommendations emphasized the importance of energy audits, sustainable building standards, reducing petroleum dependence in EMS, and promoting carbon literacy among clinicians and administrators.^12^,^13^ At the policy level, international reviews highlighted the U.S. Department of Health and Human Services (HHS) sustainability plans and WHO calls for climate-smart, low-carbon healthcare facilities.^9^ Across these studies, no papers reported the existence of a fully climate-smart ED in practice, and no empirical data quantified reductions in ED-specific carbon footprints.^9^,^12^,^13^

### Climate Resilience Interventions

All seven studies described resilience interventions relevant to EDs.^9^–^15^ Reported domains included disaster preparedness, surge capacity, infrastructure continuity, clinical protocols, and surveillance systems. Preparedness and surge planning were recurrent themes. In Tanzania, a national survey of 25 hospitals (2012) reported that only 20% had disaster plans, fewer than half had intensive care unit (ICU) capacity, and none met all surge capacity criteria. In Sri Lanka, a 2010 survey of 31 public health facilities found that only 13% had disaster plans. In Vietnam, a 2009 hospital survey indicated that more than 80% of health workers reported inadequate disaster training.^10^ Infrastructure vulnerabilities were noted across several contexts. Studies from Ghana and Nigeria described unstable electricity supply, inadequate drainage and sanitation, poor ventilation, and physical access barriers as key threats to continuity of care during climate-related events.^14^

Clinical tools and protocols were introduced in two studies. Nicholas et al developed a mnemonic tool—A CLIMATE (Act, Consider, Learn, Implement, Manage, Act, Treat, Evaluate/Educate)—to guide ED climate-related assessment and management, while Sorensen et al provided clinical practice recommendations for managing heat illness, respiratory disease, cardiovascular disease, and disaster-related trauma.^11^ Surveillance and early warning systems were also highlighted. In the U.S., New York City’s syndromic surveillance system uses ED triage data to detect heat-related illness, while the Phoenix, AZ, forecast office of the National Weather Service issues pre-heat alerts to case managers and first responders.^11^ In Africa, the Global Rural-Urban Mapping Project (GRUMP) mapping was proposed as a tool to assess flood and landslide risks, and the WHO Strategic Tool for the Analysis of Risk (STAR) was recommended for structured disaster preparedness planning.^11^,^13^,^14^

### Use of Frameworks

A range of global, regional, and ED-specific frameworks were referenced across the included studies. Global health frameworks included the WHO climate-resilient health systems framework and the Sendai Framework for Disaster Risk Reduction,^9^,^10^,^14^ both cited in reviews of emergency care in low- and middle-income countries and African contexts. Regional and national tools included the HHS Sustainable and Climate-Resilient Healthcare Facilities Toolkit in the U.S., as well as municipal- and regional-level strategies such as the Accra Climate Action Plan in Ghana. Major sustainability initiatives are also underway within the British NHS. The NHS has launched a comprehensive decarbonization strategy, “Delivering a Net Zero National Health Service,”^8^ outlining a nationwide approach to reducing healthcare-related emissions. As part of this work, some regions have begun transitioning ambulance fleets to electric, zero-emission vehicles. In the Hillingdon region of suburban London, all patient-transport ambulances have been replaced with electric vehicles,^16^ and Yorkshire Ambulance Service has begun introducing electric ambulances as part of its broader sustainability plans.^17^

These developments represent emerging examples of health-system decarbonization efforts with implications for emergency and prehospital care. African studies also referenced WHO STAR, GRUMP, and broader adaptation measures in the United National Framework Convention on Climate Change adaptation measures.^9^,^11^,^14^ Less common are ED-specific frameworks.^14^ As referenced above, Nicholas et al introduced the A CLIMATE tool for use in the ED to guide clinical assessment and management of patients affected by climate-sensitive exposures.^15^ Across all studies, frameworks were described in relation to emergency medicine but were not reported as being fully operationalized within an ED.^9^–^15^

### Opportunities for Future Development

The included studies identified several areas where future efforts could enhance ED sustainability and resilience. Research priorities were emphasized across the literature, particularly the need for more empirical studies evaluating ED-specific interventions. This gap was consistently noted as a barrier to advancing evidence-based practice in climate resilience and sustainability. Operational improvements included the development of standardized preparedness checklists and the implementation of simulation-based disaster training programs. Authors also recommended incorporating sustainability and resilience indicators into routine ED quality improvement processes.^9^,^10^,^14^ Systems integration was another recurrent theme, with calls for stronger partnerships between EDs, hospital operations, public health agencies, EMS, and community services to improve coordination and preparedness across the health systems.^11^,^13^,^14^ Equity considerations were consistently highlighted, with repeated recommendations to strengthen protections for children, older adults, pregnant persons, and socially marginalized populations that face disproportionate risks from climate-related events.^9^,^11^,^13^

## DISCUSSION

This review represents, to our knowledge, the first synthesis of literature examining sustainability and climate resilience interventions relevant to EDs. Across seven included studies, we found increasing recognition of the ED’s dual role as a major contributor to health system environmental impacts and as a frontline responder to climate-related health threats. While the literature describes a wide range of potential strategies, most reports remain conceptual, highlighting a persistent gap between frameworks and actual implementation in ED settings.

### Sustainability Interventions

Evidence of environmental footprint reduction in EDs was largely limited to hospital- or EMS-level examples rather than department-specific programs. Reports of waste reduction, device reprocessing, and fleet emission reduction illustrate the feasibility of health-sector mitigation and demonstrate both environmental and financial co-benefits.^12^,^13^ However, no studies describe the existence of a “climate-smart” ED in practice nor do any describe data-quantified ED-specific carbon footprints.^9^,^12^,^13^ This gap is significant given the energy-intensive nature of emergency care and the growing imperative for health systems to reduce GHG emissions. For broader context, healthcare systems are responsible for 4–5% of global GHG emissions,^18^ with the U.S. health sector producing 8.5–10% of total U.S. emissions, reaching 1,692 kilograms per capita in 2018, the highest rate among industrialized nations.^4^,^5^ Moving forward, ED-level carbon accounting and evaluation of targeted sustainability interventions will be essential for translating conceptual recommendations into measurable practice.

### Climate Resilience Interventions

All included studies emphasized resilience, underscoring that EDs are critical nodes of health system preparedness and continuity during climate-related events. Findings consistently revealed deficits in hospital disaster planning and surge capacity, particularly in low- and middle-income countries. Rublee et al reported that only 20% of hospitals in Tanzania had disaster plans and fewer than half had ICU capacity, while in Sri Lanka only 13% of hospitals had disaster plans and most respondents lacked disaster training.^10^ In Vietnam, more than 80% of health workers had not received formal disaster preparedness education.^10^ Even in high-resource settings, infrastructure vulnerabilities such as unstable power supply, inadequate ventilation, and supply chain disruptions were highlighted as risks to ED continuity.^11^,^13^ Clinical preparedness tools, such as the mnemonic A CLIMATE^15^ and condition-specific care pathways,^11^ demonstrate early steps toward integrating climate considerations into everyday ED practice. Similarly, examples of syndromic surveillance in New York City and pre-heat alerts in Phoenix illustrate the potential of linking ED data with early-warning systems.^11^,^13^ Yet these efforts remain fragmented, and systematic evaluations of their effectiveness are lacking.

### Framework Utilization

The literature frequently cited global and national frameworks including the WHO climate-resilient health systems framework, the Sendai Framework for Disaster Risk Reduction, and the HHS Sustainable and Climate-Resilient Healthcare Facilities Toolkit as reference points for action.^9^–^11^,^14^ Municipal and regional planning tools, such as the Accra Climate Action Plan, were also described.^14^ Nicholas et al introduced the only ED-specific framework, A CLIMATE, as a tool to guide clinicians in integrating climate considerations into patient care. The acronym stands for “A—Act immediately to stabilize life- and limb-threatening conditions; C—Consider the climate-and-health etiology of symptoms; L—Learn from a climate health history; I—Implement a climate- and health-focused assessment; M—Manage the ongoing care of the climate-related emergency; A—Act to integrate an action plan that includes physiological and psychological climate symptoms; T—Treat urgent climate symptoms and consequences; and E—Evaluate, educate, and refer for long-term follow-up.”^15^ Across studies, frameworks were referenced but not reported as being fully operationalized within ED settings. For context, updated versions of these frameworks continue to emphasize health system decarbonization and resilience.^19^

### Opportunities for Development

Several recurring themes emerged across studies as opportunities for advancing ED sustainability and resilience. Research priorities include the need for empirical studies that evaluate ED-specific interventions, as the current evidence base is dominated by conceptual and review papers.^10^,^13^–^15^ Operational recommendations emphasize standardized preparedness checklists, simulation-based disaster training, and the incorporation of sustainability and resilience indicators into ED quality improvement processes.^9^,^10^,^14^ Systems-level integration was identified as crucial, with calls for closer collaboration between EDs, hospital operations, public health, EMS, and community services.^11^,^13^,^14^ Finally, equity considerations were consistently highlighted, with recognition that children, older adults, pregnant persons, and socially marginalized groups face disproportionate risks during climate-related.^9^,^11^,^13^,^14^

### Implications for Emergency Medicine

The findings of this review suggest that emergency medicine is at an early but pivotal stage in dealing with climate change. Emergency departments have been described as experiencing “a large burden” from climate change through their focus on urgent and emergency care, their role as a safety net for vulnerable populations, and their leadership in disaster medicine.^11^ Yet the specialty has not yet translated this recognition into widespread operational change. As health systems pursue decarbonization goals and prepare for increasing climate shocks, EDs have a unique opportunity to serve as both test sites and exemplars of climate-smart care.^12^ This will require integrating environmental sustainability into departmental quality initiatives, adopting resilience metrics as part of preparedness processes, and ensuring that equity remains central to all climate and health strategies.

## LIMITATIONS

This scoping review has several limitations. First, the number of eligible studies was small, and the evidence base remains limited in both scope and depth. Most included papers were conceptual or review-based rather than empirical, and only a few provided primary data from hospital preparedness surveys. Thus, our synthesis reflects proposed frameworks and recommendations more than evaluated interventions. Second, while we sought to capture ED-specific initiatives, much of the available literature reported on hospital- or EMS-level strategies, and no study provided quantitative data on ED-specific carbon footprints. Third, the included studies varied widely in geographic focus, methodology, and level of detail, which limited direct comparison across settings. Fourth, although we conducted a systematic search across major databases, relevant articles may have been missed, particularly those published in the gray literature or in languages other than English.

Additionally, many of the included studies predated recent policy developments, such as the 2023 WHO Operational Framework for Climate-Resilient and Low-Carbon Health Systems and updated global frameworks from the HHS. Because many of the included studies were published more than five years ago, it is possible that additional ED-level sustainability and climate-smart interventions have been implemented more recently but have not yet appeared in the peer-reviewed literature. The existence of these newer frameworks suggests that efforts to reduce the GHG impact of ED operations may already be underway but remain unreported. Although this review primarily focused on peer-reviewed evidence, emerging national-scale sustainability efforts, such as those currently being implemented within the British NHS, including the introduction of electrically powered, renewably powered ambulances, indicate that system-wide changes in emergency and prehospital care are ongoing but incompletely captured in published research.

These developments also highlight differences in health-system organization across countries. In the United Kingdom, the NHS operates a unified national EMS system, which may facilitate large-scale transitions to low-emission emergency transport. In contrast, the fragmented and locally governed EMS structure in the U.S. may pose barriers to similar implementation efforts. Additionally, because more recent initiatives may not yet be published, the true extent of current ED sustainability and resilience practices may be underestimated. These limitations highlight the need for more empirical research on ED-specific interventions and the development of standardized outcome measures to guide climate-smart healthcare initiatives.

## CONCLUSION

This scoping review represents one of the first examinations of sustainability and climate resilience initiatives specific to EDs. Our findings reveal a striking paradox: While EDs are both major contributors to healthcare’s environmental footprint and frontline responders to climate-related health threats, there exists a profound gap between conceptual frameworks and operational implementation. Despite recommendations from global health organizations and the existence of multiple climate-resilience frameworks, we identified no studies documenting fully implemented climate-smart EDs. This is particularly concerning given that healthcare systems contribute 4–5% of global greenhouse gas emissions, with emergency care representing one of the most resource-intensive components of hospital operations. In the U.S., this impact is more pronounced, with healthcare responsible for an estimated 8.5–10% of national greenhouse gas emissions. far exceeding the global average and underscoring the urgency for EDs to adopt and operationalize mitigation strategies. The literature consistently describes what EDs should do to address climate change but provides no evidence of what they are doing.

The climate and health crisis demands immediate action from the specialty of emergency medicine. Emergency departments occupy a unique position as both environmental contributors and essential responders in climate disaster response. This represents an opportunity for emergency medicine to lead healthcare’s climate response while improving patient outcomes and reducing operational costs. The co-benefits documented in hospital-level interventions—such as the 31% reduction in ambulance CO_2_ emissions and $3 million savings from device reprocessing programs—suggest significant potential returns on investment.

Emergency medicine now stands at a pivotal moment. The frameworks exist, the urgency is clear, and the opportunity for impact is large. What is needed is the translation of recommendations into practice through research, standardized outcome measures, and systematic implementation of ED-specific interventions. Future efforts must focus on evaluation of sustainability initiatives, development of climate-resilience metrics, and integration of equity considerations into all climate and health strategies.

The time for conceptual discussion has passed. Emergency departments must now move beyond frameworks to become active implementers of climate-smart healthcare practices. This shift from theory to practice is critical for addressing both patient health outcomes and environmental impact.
